# Quantifying Projected Heat Mortality Impacts under 21st-Century Warming Conditions for Selected European Countries

**DOI:** 10.3390/ijerph14070729

**Published:** 2017-07-05

**Authors:** Vladimir Kendrovski, Michela Baccini, Gerardo Sanchez Martinez, Tanja Wolf, Elizabet Paunovic, Bettina Menne

**Affiliations:** 1 WHO European Centre for Environment and Health, Platz der Vereinten Nationen 1, 53113 Bonn, Germany; sanchezmartinezg@who.int (G.S.M.); wolft@who.int (T.W.); paunovice@who.int (E.P.); menneb@who.int (B.M.); 2 Department of Statistics, Informatics, Applications, University of Florence, 50134 Florence, Italy; baccini@disia.unifi.it

**Keywords:** heat-related mortality, climate change, heatwave preparedness

## Abstract

Under future warming conditions, high ambient temperatures will have a significant impact on population health in Europe. The aim of this paper is to quantify the possible future impact of heat on population mortality in European countries, under different climate change scenarios. We combined the heat-mortality function estimated from historical data with meteorological projections for the future time laps 2035–2064 and 2071–2099, developed under the Representative Concentration Pathways (RCP) 4.5 and 8.5. We calculated attributable deaths (AD) at the country level. Overall, the expected impacts will be much larger than the impacts we would observe if apparent temperatures would remain in the future at the observed historical levels. During the period 2071–2099, an overall excess of 46,690 and 117,333 AD per year is expected under the RCP 4.5 and RCP 8.5 scenarios respectively, in addition to the 16,303 AD estimated under the historical scenario. Mediterranean and Eastern European countries will be the most affected by heat, but a non-negligible impact will be still registered in North-continental countries. Policies and plans for heat mitigation and adaptation are needed and urgent in European countries in order to prevent the expected increase of heat-related deaths in the coming decades.

## 1. Introduction

Climate change is already contributing as one of the most relevant environmental risk factors to 1.4 million premature deaths in World Health Organization European Region (WHO/Europe). The direct and indirect impacts of emerging risks, such as climate change, need to be tackled urgently, as they are set to become the most challenging risks populations will face in the coming decades [1]. 

The Paris Climate Agreement provides the framework for future international cooperation and national action on climate change at the international level. Climate change and health is a central issue in the actual political discussion and projecting its impact on environment, ecosystems and human health is of great interest to inform policies and actions aimed to limit global warming and to contain its effects [2]. 

High ambient temperatures have a significant impact on society and population health, including a rise in morbidity and mortality [3]. In particular, episodes of heat lasting for several days, often referred as heat waves, were the deadliest extreme weather events in the period 1991–2015 in the European Region, causing tens of thousands of premature deaths. Under anticipated future warming conditions the length, frequency and intensity of heat waves are expected to increase and, taking in consideration that the European population is projected to age, this can lead to an important increase of heat-attributable deaths, unless effective adaptation measures are taken [4,5,6]. This raises the issue that, in order to maintain an acceptable quality of life for the foreseeable future, urban areas have to be properly managed and major actions regarding heat and heat waves and their impact on urban population have to be adopted [7], e.g., health heat action plans or heat-health warning systems [8,9].

Countries are at different stages of preparing, developing and implementing climate change and health adaptation strategies. Several Member States in WHO/Europe introduced heat wave early warning systems with heat health action plans as an approach to reducing the human health consequences of heat waves. Early warning systems predict possible health events by involving forecasting of the heat wave outcomes, and consist in timely response heat health action plans which targeting vulnerable populations [10]. Their development depends on the magnitude and nature of the observed health effects, the assessment of current and future vulnerability, the capacity to adapt, and the willingness to act [11].

In 2014, of 53 member states of the WHO European Region, 18 had developed heat-health action plans. The plans were heterogeneous in terms of spatial coverage, measures taken and existence of evaluation procedures In particular, only two of them included evaluation steps, so that detecting measures which effectively reduce heat-related mortality and morbidity remains a challenge [9]. Boeckmann and Rohn [12], on the basis of a literature review including 30 articles investigating the beneficial effect of implementing heat warning systems, concluded that these studies did not provide evidence on the efficacy of planned adaptation measures. Toloo et al. [13], in a systematic review of fifteen articles, claim for the need of further research in this field and the development of prospective designs aimed to establish whether heat warning systems can produce the expected health benefits, in particular on identified vulnerable groups.

Several studies focused on the impact of high ambient temperatures and exceptional episodes of heat for intensity and duration on mortality and morbidity of urban populations [14,15,16,17]. Recently, Sanchez et al. [18] estimated the actual and future impacts of heat on mortality in the urban area of Skopje under different climate models by combining climate and population projections, while Hunt et al. demonstrate that climate projections can be used to derive quantitative estimates of both the costs and benefits of policies aimed to reduce climate-related risks [19]. 

Europe in particular emerges as an especially responsive area to temperature rise where the warming will be larger than the projected global average increase. The strongest warming is projected across North-Eastern Europe and Scandinavia in winter and across Southern Europe in summer [20,21]. The aim of this paper is to estimate the country-specific heat mortality in selected European countries through their exposure to summertime heat under different climate change scenarios corresponding to different levels of greenhouse gas (GHG) emissions.

## 2. Materials and Methods

Our analysis focused on the following European countries: Albania, Austria, Belarus, Belgium, Bosnia and Herzegovina, Bulgaria, Croatia, Cyprus, Czech Republic, Denmark, Estonia, Finland, France, Germany, Greece, Hungary, Iceland, Ireland, Italy, Latvia, Lithuania, Luxemburg, Montenegro, Netherlands, Norway, Poland, Portugal, Republic of Moldova, Romania, Serbia, Slovakia, Slovenia, Spain, Sweden, Switzerland, The former Yugoslav Republic of Macedonia, United Kingdom of Great Britain and Northern Ireland, and Ukraine.

In our analysis we considered baseline climate (1971–2000) and meteorological projections for the future time laps 2036–2064 and 2071–2099. We focused on the Representative Concentration Pathways (RCP) 4.5 and 8.5, which stabilize radiative forcing in the year 2100 at 4.5 and 8.5 Watts per square meter, respectively. While RCP 4.5 assumes that climate policies are invoked to achieve the goal of limiting greenhouse gas (GHG) emissions, RCP 8.5 is characterized by future high energy demand and GHG emissions in absence of climate change policies. Compared to the total set of RCPs, RCP 8.5 corresponds to the pathway with the highest GHG emissions [22]. Projections were obtained according to the SMHI RCA4/HadGEM2 ES r1 (MOHC) climate model (Table 1). Regarding the historical period, we used meteorological data arising from RCP 4.5 and global climate model KNMI RACMO22E [23].

Meteorological projections spanned a 25 km × 25 km grid over the European domain and consisted in daily time series (one for each grid cell) of several meteorological indicators. In this work, we focused only on daily average dry bulb temperatures (T) and relative humidity (RH), that we combined to obtain daily mean apparent temperatures (AT) according to the formula proposed by Patricola and Cook [24]. 

### 2.1. Population and Mortality Rate Projections

Population was assumed to change over time according to the Shared Socioeconomic Pathway SSP2, a median population growth scenario that combines for all countries medium fertility with medium mortality, medium migration and some moderate expansion in education [25]. According to this scenario, world population is expected to peak around 2070. For each country, the overall population mortality during the two future time slices was obtained by using the overall crude mortality rate projected under the assumption of median fertility [26]. 

### 2.2. Heat-Mortality Function

In order to estimate the heat-mortality relationship, we used the city-specific heat-mortality functions estimated for the 15 European cities participating in the “Assessment and Prevention of acute Health Effects of Weather conditions in Europe” (PHEWE) project [27]. These functions refer to the effect of maximum apparent temperature on natural mortality (all ages) during the 90 s and are summarized by a threshold, corresponding to the minimum of the heat-mortality curve, and a slope above this threshold. In order to account for geographical heterogeneity, we performed a Bayesian meta-analysis of these city-specific thresholds and slopes, separately for Mediterranean cities, Northern continental cities and Eastern cities. While random effects models were specified for Mediterranean and Northern continental cities, a fixed effects model was used for Eastern cities, because, being the meta-analysis performed on only two results, a reliable and stable estimate of the between study variance could not be obtained. In Table 2 the posterior distributions of the overall meta-analytic slopes and thresholds are summarized in terms of means and 90% credibility intervals (5th and 95th percentiles of the posterior distributions). Slopes are reported as percent change in mortality associated to 1 °C increase of maximum apparent temperature above the threshold. We calculated also the I^2^ index for each random effects meta-analysis, which expresses the percentage of total variance explained by differences among cities.

Due to the PHEWE results used in our meta-analyses refer to maximum apparent temperatures, we applied ad hoc conversion terms to adapt them to mean apparent temperature. In order to estimate the conversion terms, we specified a simple regression model to compare daily maximum apparent temperature (outcome variable) and daily mean apparent temperature (explanatory variable) in each of the 15 cities enrolled in the PHEWE project. All the regression slopes resulted to be approximately equal to 1, suggesting that adjustment for the slope above the threshold was not needed. On the contrary, the estimated intercepts were different from 0; thus, we averaged them separately by region to obtain conversion constants to be subtracted from the maximum apparent temperature thresholds. Table A1 reports the constant terms arising from this procedure as well as the initial thresholds and the final mean apparent temperature thresholds.

### 2.3. Attributable Fraction at the Cell Level

For each grid cell *i* and each day *d* belonging to the “warm season” of the year *y* (April–September, for a total of 183 days), the daily attributable fraction (*AF_idy_*) (i.e., the fraction of deaths attributable to mean apparent temperature above the threshold) was calculated according to the following equations:

*AF_idy_* = 1 − 1/exp(*b_i_*(*AT_idy_* − *h_i_*))   if *AT_idy_* > *h_i_* *AF_idy_* = 0   if *AT_idy_* ≤ *h_i_* (1)

where *AT_idy_* was the mean apparent temperature, *h_i_* was the threshold (Table 3), and *b_i_* was the slope above the threshold for the cell *i* (reported in terms of % variation in Table 2). Then, we calculated the average AF during each warm season *y* for each cell of the map:

(2) $${\bar{AF}}_{iy}=\underset{d\in y}{\sum }AF_{idy}/183$$

This value was used as a relative measure of the “overall” impact of heat on mortality during *y* to allow comparisons among cells/areas. For simplicity, in the formulas we suppressed the reference to the RCP scenario, even if each indicator was calculated for each of the different combinations in Table 1.

### 2.4. Country-Level AF

In order to estimate AFs at the country level, we averaged ${\bar{AF}}_{iy}$ over the *n_c_* cells belonging to the same country *c*. We did it separately for each year so that we got annual time series of AFs for each country:

(3) $${\bar{AF}}_{cy}=\underset{i\in c}{\sum }{\bar{AF}}_{iy}/n_{c}$$

In order to represent the annual variability of the impact, the minimum, maximum and average AFs over each time slice were calculated for each country. Finally, we averaged ${\bar{AF}}_{cy}$ over years within each time slice *p* to calculate the average AF for country *c* during time slice *p* ${\bar{AF}}_{cp}$:

(4) $${\bar{AF}}_{cp}=\underset{y\in p}{\sum }{\bar{AF}}_{cy}/n_{p}$$

where *n_p_* was the number of years in *p*.

### 2.5. Expected Attributable Deaths (AD)

For the future time slices 2036–2064 and 2071–2099, we estimated the number of deaths attributable to daily apparent temperatures above the comfort threshold (AD) according to the following approximated formula:

(5) $$AD_{cp}={\bar{AF}}_{cp}\times pop_{cp}\times r_{cp}\times \lambda$$

where *pop_cp_* was the average population of country *c* during time slice *p* from SPP2 [21]; *r_cp_* was the average annual crude mortality rate for *c* during *p*; *λ* was an estimate of the proportion of annual deaths observed during the warm season, estimated from the original PHEWE data sets (*λ* = 0.45).

For each country, time slice and RCP scenario, the attributable community rate (ACR), corresponding to the ratio between AD and population size, was also calculated (Table A2). We also evaluated the impact of climate change taking as reference the historical meteorological conditions observed during the period 1971–2001. In order to do this, we estimated counterfactual ADs by introducing in Equation (5) the historical AFs instead of the projected ones. These counterfactual ADs measured the impact that we would observe during the future time slices if apparent temperatures would remain unchanged at the levels measured during the reference period. Then, we compared these impact estimates with those obtained under RCP 4.5 and RCP 8.5 scenarios, thus quantifying the number of deaths that could be prevented maintaining apparent temperatures at their past levels. 

## 3. Results

The country-specific attributable fractions (AFs) estimated for the historical period and projected for the future time slices under the two Representative Concentration Pathways are reported in Table 3. According to these results, the percentage of deaths attributable to heat is expected to increase over time.

The estimated AFs are heterogeneous across the selected European countries, in particular when considering the time period 2071–2099 and the RCP 8.5 scenario. 

According to our results, the inter-quartile range (IQR) of the country-specific percent AFs is expected to be (0.20; 1.36) in 2039–2064 and (0.38; 1.84) in 2071–2099 under RCP 4.5. The same IQRs under RCP 8.5 are expected to be even wider: (0.49; 1.88) in 2039–2064 and (1.18; 5.22) in 2071–2099. These values should be compared with the IQR of the historical AFs which range between 0.05% and 0.46%. 

**Figure 1:**
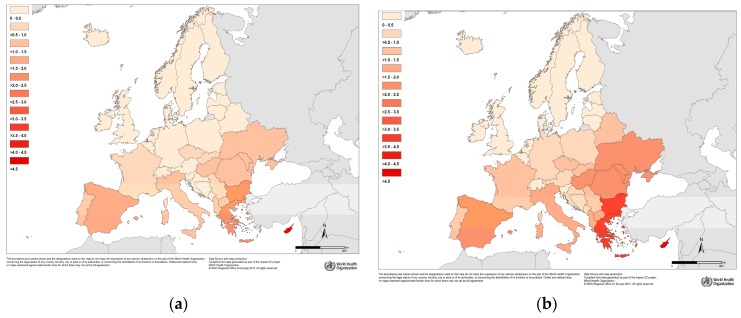

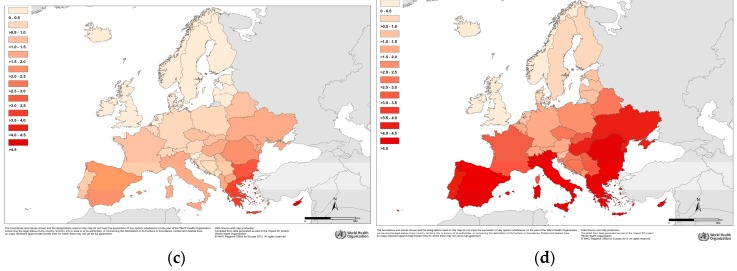
Attributable fraction (AF) of heat-related deaths during summer by country in European sub-region in 2050: (**a**) Using MOHC model in RCP 4.5; (**b**) Using MOHC model in RCP 8.5, and in 2085: (**c**) Using MOHC model in RCP 4.5; (**d**) Using MOHC model in RCP 8.5.

In Figure 1 the average AFs are reported for the years 2050 and 2085 (${\bar{AF}}_{c2050}$; ${\bar{AF}}_{c2085}$) by country, under both scenarios. 

The map, which focuses on the central years of the time laps, clearly shows that the average AFs during summer are expected to be higher in Mediterranean and Eastern European countries than in the rest of Europe. However, there will be still heat-related deaths in the North-Continental region in particular under RCP 8.5. 

The inter-annual variability of AFs is summarized in Table A3, where the minimum, maximum and average AFs over each time slice are reported for each country under RCP 4.5 and RCP 8.5.

Table 4 shows the Attributable deaths (AD) calculated assuming as counterfactual the impact that we would observe in the future time slices assuming that the AFs would remain at the levels observed during the historical period 1971–2000. 

Overall, 30,867 more AD per year are expected during the period 2036–2064 under RCP 4.5 and 45,930 under RCP 8.5, taking as reference the impact that we would observe if the apparent temperatures would remain at the historical levels observed during the time slice 1971–2001 (17,384 AD). The estimated impacts are much larger for the period 2071–2099: 46,690 and 117,333 AD per year under RCP 4.5 and RCP 8.5, respectively, in addition to the 16,303 AD expected under the reference. 

**Figure 2:**
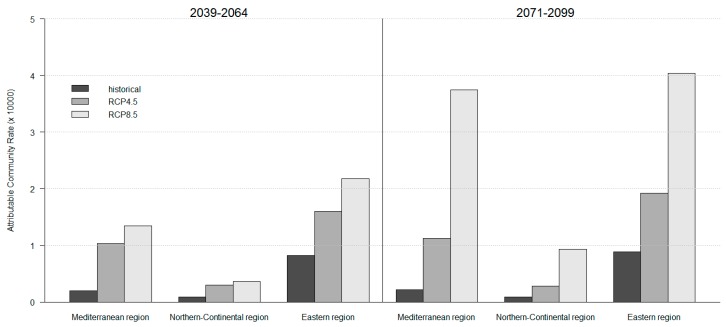
Attributable Community Rate (ACR) per 10,000 inhabitants expected for the future time laps 2036–2064 and 2071–2099 under the historical scenario, RCP 4.5 and RCP 8.5 by macro-region (RCP: Representative Concentration Pathways).

Figure 2 shows the overall ACRs per 10,000 inhabitants estimated for the two future time slices under the historical scenarios and the RCP 4.5 and RCP 8.5 scenarios, separately by macro-region. While in Northern-Continental countries ACR is expected to remain under 1 AD per year per 10,000 inhabitants, warming will have major consequences in Mediterranean and Eastern Europe where ACRs could exceed 3 ADs per 10,000 during the period 2071–2099 under RCP 8.5.

With reference to Table 4, Table A2 and Figure 2, it is worth noticing that under the reference scenario, which assumes stable climate conditions in respect to the past, impact variations over time only reflect changes in mortality rate and population size.

## 4. Discussion

Current high summer ambient temperatures have an important impact on the European population health and increase of heat-related mortality and morbidity are identified as being a potentially significant consequence of climate change in Europe [28,29]. This impact is expected to increase in the future, according to the projected increase of mean ambient temperatures [30]. Several studies estimated the future heat-related mortality in European Union countries arriving at largely comparable results: Projection of Economic impacts of climate change in Sectors of the European Union based on bottom-up Analysis (PESETA), ClimateCost and PESETA II [31,32,33,34].

In this paper we provide an overall picture of the possible future impact of heat on population mortality in Europe, under different climate change scenarios corresponding to different levels of greenhouse gas emissions. We considered selected climate model RCP 8.5 and RCP 4.5. In our study summer temperatures (April-September) are shown to influence daily mortality across Europe due to climate change impacts. We found that heat impacts will dramatically increase over time, in particular under the RCP 8.5 scenario and in Mediterranean and Eastern European countries. These regions are in fact expected to face the continent's most adverse effects from climate change as heatwaves and droughts become more intense and frequent [20,21].This result is in line with the literature (see for example the PESETA II study [33] and with the estimates produced by the World Health Organization (WHO) for the WHO European Region [35]. The impact of heat, although lower, will not be negligible for the citizens in North-Western European countries too.

It is worth to notice that our results do not account for harvesting phenomena acting by anticipating deaths in frail subpopulation. This does not invalidate our estimates, but it should be stressed that studying the mechanisms underlying heat effects and impacts on health is a priority to provide useful evidence to inform policies and interventions on the population [14]. 

Our study has several limitations that should be accounted for in interpreting the reported results. According to several studies conducted in Europe and elsewhere, in the last 20 years population susceptibility to heat and heatwaves decreased. Improvements in infrastructures and health care services, together with the implementation of heat-adaptation measures and heat health watch warning systems, likely contributed to improve population adaptation, reducing the impact of heat on mortality and morbidity [36,37,38,39,40,41]. However, few studies attempt to quantitatively attribute changes in susceptibility to specific adaptive measures, and estimating the effect of specific heat adaptation plans on population health remains a challenge, as well as characterizing and predicting how the relationship between heat and health outcomes varies over time [9,10]. A further difficulty is related to the heterogeneity of the susceptibility decrease across locations. For example, De’ Donato et al. [42] compared the mortality risk associated to heat in nine European cities before and after summer 2003, finding a reduction of susceptibility only in Athens, Rome and Paris. For these reasons, despite the relevance that a decrease of heat-related susceptibility may have in predictions, we use the historical heat-mortality functions to estimate future impacts, without considering possible acclimatization or adaptation mechanisms that could mitigate the effect of heat in the future. 

We do not consider the additional effect of heat waves [16], possibly underestimating the impact of warming. Moreover, our results are obtained assuming that a uniform population distribution within each country. Considering that urban areas are more densely populated than rural ones and usually characterized by higher ambient temperatures, even this assumption could have brought to underestimating future impacts, especially for those countries where urbanization is expected to increase over time. 

We adopt an ad hoc approach, which allows us to use the slope estimated for maximum apparent temperature as a surrogate of the slope above the threshold for mean apparent temperature. This procedure, that appears to be appropriate in our specific context, could in general introduce a certain amount of bias in the results and should not be applied without careful check of daily data. 

Despite population is ageing in many European cities, thus potentially inflating the pool of subjects vulnerable to heat [43,44], our analyses are conducted without accounting for the age distribution of the population, possibly underestimating the number of attributable deaths in the future. Finally, due to the complexity of the data set, we did not account for uncertainties related to heat-mortality functions, population projections and mortality rates. A partial evaluation of the uncertainty of the phenomenon can be obtained by considering the inter-annual variability of climate projections. 

## 5. Conclusions

Advocating for further development and implementation of heatwave planning, preparedness, and response in European countries would lead to a reduction in heat-related mortality. Strong inter-sectoral coordination and cooperation as well as surveillance and evaluation measures in place through an effective early warning and health system response mechanisms are desirable targets [9]. Long-term regulatory planning, including urban planning, infrastructure and housing, becomes even more relevant than before.

The direct and indirect impacts of emerging risks, such as heat waves due to climate change, need to be tackled urgently, as they are set to become the most challenging risks populations in Europe will face in the coming decades. It should be continuing regional support and coordination for health components of national adaptation planning processes and adaptation actions, and through prioritizing mitigation actions that also improve health. This article presented and discussed the future impact of heat on population mortality in Europe, by country, under different climate change scenarios. Both, citizens from Mediterranean and Eastern European countries will be most affected by heat. This is an important opportunity to enhance the effective use of health projections by public health professionals, to plan policies and actions and state intervention priorities.

## Figures and Tables

**Table 1:** Study models *.

Scenario	Climate Model	Temperature/Precipitations	Historical Period (1971–2000)	2036–2064	2071–2099
RCP 8.5	SMHI RCA4/HadGEM2 ES r1 (MOHC)	Hottest/Wettest		X	X
RCP 4.5	SMHI RCA4/HadGEM2 ES r1 (MOHC)	Hottest/Wettest		X	X
RCP 4.5	KNMI RACMO22E/EC EARTH r1	Mid/Mid	X		

RCP: Representative Concentration Pathways; * All models we used were available for IMPACT2C partners [23].

**Table 2:** Summaries of the posterior distributions of threshold, % variation associated to 1 °C increase in maximum apparent temperature above the threshold and I^2^ index for Mediterranean, Northern-continental and Eastern countries.

Region	Threshold (°C) (Posterior Mean and 90% CrI)	I^2^ (Posterior Median and 90% CrI)	% Variation (Posterior Mean and 90% CrI)	I^2^ (Posterior Median and 90% CrI)
Mediterranean countries	29.4 (26.5, 31.8)	75% (43%, 94%)	3.09 (1.09, 5.07)	97% (94%, 99%)
Northern Continental countries	23.8 (23.2, 24.4)	0% (0%, 28%)	1.79 (−0.34, 3.94)	80% (60%, 94%)
Eastern countries	22.6 (21.9, 23.3)		1.77 (1.57, 1.97)	

I^2^: percentage of total variance explained by differences among cities; CrI: Credible interval.

**Table 3:** Expected fractions of deaths attributable to mean apparent temperatures above the comfort threshold, evaluated over the historical period 1971–2000 and over the future time slices 2036–2064 and 2071–2099 under the RCP 4.5 and RCP 8.5 scenarios.

Country	1971–2000	2036–2064		2071–2099	
	Historical AF (%)	RCP 4.5 AF (%)	RCP 8.5 AF (%)	RCP 4.5 AF (%)	RCP 8.5 AF (%)
Albania	0.15	1.25	1.84	1.81	5.15
Austria	0.14	0.56	0.70	0.78	1.96
Belarus	0.50	0.89	1.55	1.64	3.02
Belgium	0.14	0.43	0.58	0.70	1.70
Bosnia and Herzegovina	0.04	0.47	0.73	0.74	2.65
Bulgaria	2.02	4.49	5.55	5.44	8.82
Croatia	0.10	0.56	0.81	0.86	2.66
Cyprus	2.11	7.11	9.21	9.39	15.44
Czech Republic	0.44	1.18	1.48	1.62	3.39
Denmark	0.05	0.19	0.29	0.34	0.88
Estonia	0.05	0.18	0.33	0.45	1.23
Finland	0.03	0.12	0.19	0.28	0.90
France	0.42	1.35	1.53	1.70	3.84
Germany	0.19	0.58	0.73	0.83	1.89
Greece	0.40	2.72	4.10	3.96	8.76
Hungary	1.65	3.06	3.72	3.65	6.15
Iceland	0	0	0	0	0
Ireland	0	0.05	0.08	0.09	0.37
Italy	0.23	1.41	1.84	2.07	5.24
Latvia	0.07	0.18	0.36	0.48	1.16
Lithuania	0.10	0.24	0.48	0.60	1.32
Luxembourg	0.17	0.58	0.76	0.91	2.23
Montenegro	0.05	0.41	0.65	0.65	2.39
Netherlands	0.13	0.31	0.42	0.51	1.15
Norway	0	0.03	0.06	0.09	0.34
Poland	0.46	0.96	1.35	1.40	2.74
Portugal	0.31	1.34	1.73	1.68	4.35
Republic of Moldova	1.72	3.05	4.19	3.70	6.57
Romania	1.42	2.85	3.72	3.46	6.11
Serbia	0.09	0.74	1.19	1.12	3.44
Slovakia	0.60	1.37	1.81	1.84	3.66
Slovenia	0.01	0.11	0.19	0.22	1.02
Spain	0.47	2.12	2.50	2.80	6.12
Sweden	0.01	0.06	0.11	0.17	0.59
Switzerland	0.05	0.39	0.51	0.58	1.74
The former Yugoslav Republic of Macedonia	0.09	1.29	2.03	1.89	5.25
United Kingdom of Great Britain and Northern Ireland	0.01	0.07	0.12	0.13	0.38
Ukraine	1.32	2.40	3.43	3.21	5.49

AF: Attributable fraction; RCP: Representative Concentration Pathways.

**Table 4:** Attributable deaths per warm season expected for the future time slices 2036–2064 and 2071–2099 under the reference scenario (apparent temperatures at the historical levels observed during the period 1971–2001) and additional attributable deaths in respect to this counterfactual as expected under the RCP 4.5 and RCP 8.5 scenarios, by country.

Country	2036–2064			2071–2099		
	*AD_ref_*	Additional AD in Respect to *AD_ref_*		*AD_ref_*	Additional AD in Respect to *AD_ref_*	
	No Change	RCP 4.5	RCP 8.5	No Change	RCP 4.5	RCP 8.5
Albania	24	185	283	26	303	910
Austria	71	212	285	69	317	901
Belarus	345	269	725	288	656	1452
Belgium	93	188	287	93	358	1007
Bosnia and Herzegovina	9	99	162	9	171	634
Bulgaria	1150	1401	2008	1031	1742	3463
Croatia	28	132	205	26	206	690
Cyprus	211	499	708	160	551	1009
Czech Republic	312	531	739	327	879	2199
Denmark	16	46	77	17	101	291
Estonia	4	11	24	4	32	94
Finland	8	30	54	9	84	291
France	1499	3264	3900	1572	4739	12675
Germany	1044	2142	2949	973	3286	8693
Greece	277	1631	2595	260	2346	5502
Hungary	1043	886	1305	953	1148	2587
Iceland	0	0	0	0	0	0
Ireland	0	12	20	1	27	110
Italy	857	4454	6099	808	6551	17860
Latvia	10	16	42	8	48	126
Lithuania	21	29	78	18	89	218
Luxembourg	6	14	21	9	39	108
Montenegro	2	15	26	2	26	100
Netherlands	136	190	303	134	396	1052
Norway	1	8	18	1	31	121
Poland	1148	1252	2230	1132	2308	5611
Portugal	224	748	1029	249	1095	3235
Republic of Moldova	329	253	470	193	221	544
Romania	1959	1972	3177	1639	2363	5432
Serbia	63	478	809	69	830	2684
Slovakia	237	305	479	249	517	1276
Slovenia	1	15	26	1	30	144
Spain	1474	5207	6398	1599	7987	19330
Sweden	6	25	53	6	94	345
Switzerland	21	132	180	24	246	790
The former Yugoslav Republic of Macedonia	13	176	285	14	305	871
United Kingdom of Great Britain and Northern Ireland	46	221	377	52	466	1498
Ukraine	4696	3819	7504	4278	6102	13480
Overall	17384	30867	45930	16303	46690	117333

AD: Attributable deaths; *AD_ref_*: Attributable deaths under the reference scenario.

## Appendix A

**Table A1:** Conversion terms and mean apparent temperature thresholds by European region.

Region	Maximum Apparent Temperature Threshold (°C)	Conversion Constant (°C)	Mean Apparent Temperature Threshold (°C)
Mediterranean countries	29.4	3.7	25.7
Northern-Continental countries	23.8	2.8	21.1
Eastern countries	22.6	3.3	19.3

**Table A2:** Attributable community rate by countries estimated under RCP 4.5 and RCP 8.5 for the future time laps 2036–2064 and 2071–2099.

Country	2036–2064		2071–2099	
	RCP 4.5 ACR (per 10,000 Inhabitants)	RCP 8.5 ACR (per 10,000 Inhabitants)	RCP 4.5 ACR (per 10,000 Inhabitants)	RCP 8.5 ACR (per 10,000 Inhabitants)
Albania	0.6	1.69	1.3	3.6
Austria	0.3	0.56	0.4	1.1
Belarus	0.7	1.05	1.4	2.6
Belgium	0.2	0.44	0.3	0.8
Bosnia and Herzegovina	0.3	0.69	0.7	2.6
Bulgaria	4	3.53	5.1	8.2
Croatia	0.4	0.71	0.7	2
Cyprus	4.7	7.1	4.4	7.2
Czech Republic	0.7	1.04	1.1	2.2
Denmark	0.1	0.24	0.2	0.4
Estonia	0.1	0.28	0.3	0.8
Finland	0.1	0.16	0.1	0.4
France	0.6	1.11	0.8	1.7
Germany	0.4	0.54	0.6	1.4
Greece	1.7	3.7	2.6	5.7
Hungary	2.1	2.07	2.8	4.7
Iceland	0	0	0	0
Ireland	0	0.08	0	0.2
Italy	0.9	1.61	1.3	3.4
Latvia	0.1	0.29	0.4	0.9
Lithuania	0.2	0.38	0.5	1.1
Luxembourg	0.3	0.59	0.5	1.2
Montenegro	0.3	0.6	0.4	1.6
Netherlands	0.2	0.29	0.3	0.6
Norway	0	0.06	0	0.1
Poland	0.7	0.89	1.2	2.3
Portugal	0.9	1.42	1.3	3.3
Republic of Moldova	2.5	2.47	3.1	5.5
Romania	2.2	2.3	3.1	5.5
Serbia	0.6	1.1	1.1	3.2
Slovakia	1	1.21	1.6	3.2
Slovenia	0.1	0.18	0.1	0.7
Spain	1.3	2.03	1.9	4.2
Sweden	0	0.1	0.1	0.2
Switzerland	0.2	0.46	0.3	0.9
The former Yugoslav Republic of Macedonia	0.8	1.94	1.5	4.1
United Kingdom of Great Britain and Northern Ireland	0	0.11	0.1	0.2
Ukraine	2.2	2.11	3	5.1

**Table A3:** Average, maximum and minimum attributable fractions evaluated over the future time slices 2036–2064 and 2071–2099 under the RCP 4.5 and RCP 8.5 scenarios.

Country	2036–2064						2071–2099					
	RCP 4.5			RCP 8.5			RCP 4.5			RCP 8.5		
	Average AF (%)	min	max	Average AF (%)	min	max	Average AF (%)	min	max	Average AF (%)	min	max
Albania	1.25	0.10	3.61	1.84	0.25	3.87	1.81	0.44	4.37	5.15	1.94	8.36
Austria	0.56	0.05	2.32	0.70	0.01	2.28	0.78	0.13	2.38	1.96	0.68	3.63
Belarus	0.89	0.18	2.21	1.55	0.37	3.26	1.64	0.27	3.64	3.02	0.85	4.37
Belgium	0.43	0.00	1.27	0.58	0.00	1.89	0.70	0.02	2.12	1.70	0.59	3.28
Bosnia and Herzegovina	0.47	0.00	2.62	0.73	0.00	2.18	0.74	0.04	2.86	2.65	0.35	5.75
Bulgaria	4.49	2.66	6.48	5.55	2.84	8.91	5.44	2.92	6.95	8.82	6.27	12.48
Croatia	0.56	0.03	2.62	0.81	0.01	2.72	0.86	0.07	2.95	2.66	0.60	6.20
Cyprus	7.11	5.01	9.08	9.21	6.21	13.33	9.39	7.59	11.01	15.44	12.08	18.37
Czech Republic	1.18	0.13	3.45	1.48	0.05	4.10	1.62	0.48	4.21	3.39	1.18	5.86
Denmark	0.19	0.01	0.70	0.29	0.00	0.92	0.34	0.01	0.80	0.88	0.21	2.09
Estonia	0.18	0.01	0.38	0.33	0.04	0.85	0.45	0.01	1.32	1.23	0.30	2.25
Finland	0.12	0.00	0.28	0.19	0.03	0.64	0.28	0.02	0.79	0.90	0.27	2.13
France	1.35	0.17	3.28	1.53	0.30	3.50	1.70	0.34	3.67	3.84	1.73	5.86
Germany	0.58	0.03	1.97	0.73	0.02	2.53	0.83	0.14	2.71	1.89	0.65	3.98
Greece	2.72	1.21	4.79	4.10	1.73	7.15	3.96	1.76	5.65	8.76	6.03	11.87
Hungary	3.06	1.20	5.83	3.72	0.80	7.52	3.65	1.66	6.95	6.15	3.53	9.12
Iceland	0.00	0.00	0.00	0.00	0.00	0.00	0.00	0.00	0.00	0.00	0.00	0.00
Ireland	0.05	0.00	0.30	0.08	0.00	0.29	0.09	0.00	0.46	0.37	0.06	0.93
Italy	1.41	0.50	4.05	1.84	0.27	4.09	2.07	1.02	4.16	5.24	2.63	8.48
Latvia	0.18	0.01	0.49	0.36	0.05	0.93	0.48	0.04	1.45	1.16	0.13	2.25
Lithuania	0.24	0.02	0.75	0.48	0.07	1.26	0.60	0.03	1.78	1.32	0.20	2.93
Luxembourg	0.58	0.00	2.08	0.76	0.00	2.38	0.91	0.05	2.91	2.23	0.54	4.40
Montenegro	0.41	0.02	1.97	0.65	0.02	1.67	0.65	0.07	2.49	2.39	0.41	4.52
Netherlands	0.31	0.01	0.87	0.42	0.00	1.33	0.51	0.00	1.60	1.15	0.37	2.32
Norway	0.03	0.00	0.09	0.06	0.01	0.34	0.09	0.02	0.22	0.34	0.15	0.88
Poland	0.96	0.18	2.55	1.35	0.11	3.66	1.40	0.36	3.85	2.74	0.87	4.54
Portugal	1.34	0.36	3.24	1.73	0.67	3.28	1.68	0.78	3.47	4.35	2.35	6.43
Republic of Moldova	3.05	0.97	5.02	4.19	1.34	7.95	3.70	1.11	6.46	6.57	3.70	10.03
Romania	2.85	1.44	4.59	3.72	1.35	7.13	3.46	1.49	5.61	6.11	3.64	9.05
Serbia	0.74	0.01	2.69	1.19	0.03	3.14	1.12	0.13	3.28	3.44	0.87	6.69
Slovakia	1.37	0.29	3.32	1.81	0.13	4.67	1.84	0.56	4.59	3.66	1.54	5.45
Slovenia	0.11	0.00	1.10	0.19	0.00	1.03	0.22	0.00	1.01	1.02	0.18	2.81
Spain	2.12	0.93	3.69	2.50	1.39	4.00	2.80	1.53	4.09	6.12	3.68	7.91
Sweden	0.06	0.00	0.13	0.11	0.02	0.34	0.17	0.02	0.45	0.59	0.24	1.30
Switzerland	0.39	0.01	1.91	0.51	0.00	1.96	0.58	0.02	1.84	1.74	0.47	3.42
The former Yugoslav Republic of Macedonia	1.29	0.15	3.17	2.03	0.39	4.34	1.89	0.32	3.29	5.25	2.58	8.04
United Kingdom of Great Britain and Northern Ireland	0.07	0.00	0.27	0.12	0.00	0.52	0.13	0.00	0.43	0.38	0.04	0.83
Ukraine	2.40	1.07	4.49	3.43	1.18	5.74	3.21	1.31	5.49	5.49	2.59	8.21
